# Health belief model factors as predictors of parental misclassification of the weight of the preschool child

**DOI:** 10.1371/journal.pone.0252981

**Published:** 2021-09-10

**Authors:** Tanna Woods, Mary A. Nies

**Affiliations:** 1 Nursing Education Services, Nightingale College, Salt Lake City, Utah, United States of America; 2 College of Health, Idaho State University, Pocatello, Idaho, United States of America; University of South Florida, UNITED STATES

## Abstract

**Background:**

Parental misperception and underestimation of their child’s weight are documented in studies. Demographic factors like age and gender have been linked to misclassification. However, modifiable factors that could potentially frame future intervention and prevention strategies have not been explored. This study aimed to assess factors that could predict parental misclassification of their preschool child’s weight.

**Methods:**

This was a cross-sectional study with 198 parents and their 2- to 5-year-old children who attended standalone preschools or childcare centers with preschools. Parents completed a questionnaire that asked about demographic features, personal and family health, and the assessment of their child’s weight using the three most frequently utilized measures. Logistic regression was conducted to assess the association between parental factors and child weight classification status. Instruments included the Parental Self-Efficacy for Promoting Healthy Physical Activity and Dietary Behaviors in Children Scale (PSEPAD), the Obesity Risk Scale (ORK-10), and the Adolescent Obesity Risk Scale (AORK). Analyses included frequencies, chi-square tests, Kappa coefficients, and logistic regressions.

**Results:**

Parents were least accurate (35.9%) identifying child weight when selecting a picture (κ = -.028, p = .42). The pictorial and Likert method (κ = -.032, p = .37) showed parental agreement with child weight was not significantly better than chance. Statistically, a significant agreement was found in the weight-reporting method (κ = .21). Two of the three HBM-related measures were significantly related to accurate classification. Logistic regression showed child sex, PSEPAD scores, and ORK-10 scores were statistically significant predictors in the Likert method. The model had no statistical significance for the pictorial or weight-reporting method.

**Conclusion:**

Results indicate parents support intervening if aware of child weight problems. However, parents do not accurately recognize healthy versus unhealthy weights and report that health providers are not informing them of weight deviations. Further, important relationships between the HBM variables were identified. Results show barriers (self-efficacy) mediate the impact of perceived severity (knowledge) regarding the parental ability to assess child weight accurately. These relationships and incorporation of the HBM principles of barriers and severity into prevention/intervention strategies need further exploration.

## Introduction

Weight issues among all age groups are a critical problem. In children, overweight and obesity levels are nearing epidemic proportions and are accompanied by widespread and unnecessary health and social consequences that can extend into adulthood [1].

The adaptability of preschool-aged children between ages two and five makes this timeframe critical for prevention and intervention activities because there is a higher likelihood of success [2].

While this age group is adaptable, it is also reliant on parents and their influence [3–5]. Parents are seen as gatekeepers for these children and their beliefs, behaviors, and diet [6]. Thus, parental awareness of child weight and the accompanying issues of excess weight has been noted as the key to success for intervention and prevention for preschoolers [6].

The inaccuracy of parental perception of child weight is an identified barrier confirmed by past research and linked to demographic features like child age and gender [3–5, 7–9]. If identifying childhood weight issues is indeed a first step in treating obesity as researchers have claimed, then the success of intervention and prevention could hinge on parental ability to correctly classify their child’s weight [3, 6]. However, modifiable factors that can be used to frame future intervention and prevention efforts have not been explored.

### Conceptual framework

Lifestyle modification for health improvement has been proven effective utilizing the health belief model (HBM) in areas such as vaccination use, cancer screening, physical activity, and weight loss interventions and behaviors [10–12]. Given that preschool-aged children have been identified as the optimal group for weight intervention and prevention [2] and that parents are identified as key gatekeepers for children’s beliefs, behaviors, and diet [6], this study sought to evaluate if parental factors relating to key components of HBM could be linked to prediction of the child weight. The components of the HBM are associated with readiness for behavior change, any identified connections could have potential benefits for framing prevention and intervention strategies. Specifically, this study examined severity, benefits, and barriers from the HBM as these have been deemed the most influential moderators [13].

The HBM explains individual behaviors and emphasizes the importance of perceiving conditions as severe or as a severe risk as necessary to increase the likelihood of action to counteract it [14]. Self-efficacy as a separate important factor needed for behavioral change has also been used and justified as confidence in the ability to change is important, though self-efficacy has also been considered as part of the perceived barriers factor [10].

### Study purpose

This study examined factors that can affect parental misclassification and potentially limit parental behavior changes that are necessary to correct or prevent overweight and obesity problems in their children.

## Materials and methods

### Ethical consideration

The Idaho State University Institutional Review Board approved this student. IRB-FY2018-249: Health Belief Model Factors as Predictors of Parental Misclassification of the Weight of the Preschool Child. Parents provided written consent for their participation and that of their child. Verbal assent was obtained for the children prior to the weight and height being collected.

### Sample size and power analysis

Sample size calculations were performed using G*Power: Statistical Power Analyses to determine the maximum needed samples to answer the questions. These sample size needs vary for the questions. For questions involving the point-biserial correlation, the sample size needed is 82. This would detect a point-biserial correlation of .30 or higher at alpha = .05 (two-tailed) with minimum power = .80 and thus only require a minimum sample size of 82 participants. For logistic regression, the sample depends on the final distribution for the categories of 1 = correct and 0 = incorrect. The research done in this area has wide variability for misclassification rates with some being very high. If looking at the portion of correct classifications being as low as .40 for correct and .60 for incorrect with a power .80 using a one-tailed test and having a meaningful odds ratio detected at 1.5, the sample needs to be 169. By changing the odds ratio to 1.7, the sample size would need to be 104. A larger sample size would allow an increased ability to detect a smaller effect size (odds ratio).

Given the surveyor was attempting to gain participation mainly by sending items home with students with a secondary approach of handing out surveys to parents in person, there was a consideration to ensure proper sample size was obtained. Unsolicited surveys with no face-to-face request have a 50% return rate [15]. With this in mind as well as the fact that 250 surveys were sent home to parents in children’s backpack, we anticipated that 125 or less would participate. The option of sending home surveys with children was not available at all participating centers and the investigator had to be present to address parents individually during drop-off and pick-up times. An additional 165 surveys were directly handed to parents.

As attrition can be expected, the sample goal was 106 participants to account for 30% attrition. The 30% was determined as a conservative, low number would be a 10% return while a conservative higher number could be 40%. With 30% attrition being planned for in this study, 10 recruitment sites were needed to account for varying numbers of eligible children at each location. To plan for attrition and improve the ability to detect smaller effects, the minimum sample size needed was 104 with a goal of 150 participants.

### Study design and population

A cross-sectional design was utilized to survey parents of 2- to 5-year-old children. Both standalone preschools and daycares with preschool (n = 17) were used for recruitment. Any person with legal responsibility for the child, with or without a biological connection, who performed everyday care for the child was eligible to participate as a parent [16].

Inclusion criteria included the ability to speak and read English, to be a self-identified legal guardian for the participating child who was within the age group and were willing to sign a written consent. Participants were excluded if another child of the parent had already participated or if the child had a disease that is known to affect weight/size, including pituitary and thyroid conditions. There were no restrictions based on race/ethnicity, education level, or income. It was possible that a written questionnaire could limit participation to only those who can read and write, which was an unavoidable limitation.

#### Participants and procedure

The two counties where participants were recruited included a population make-up that aligned with the state demographics. This study employed purposive, non-probabilistic, and convenience sampling to obtain a diverse sample. While the inclusion of daycares and preschools increased the chance of a more diversified sample, it still had the potential to eliminate parents who chose to homeschool or not utilize preschools.

The study was advertised by signs at the facilities and information sent home with children who fit the ages of 2 to 5. Additionally, the investigator was at the facility during drop off and pick up times to recruit parents.

Parents completed a written consent for their child’s participation in the study as well as a written survey, which they returned to a locked collection box at their facility. A trained researcher obtained verbal consent from the child (and written from the parents) then weighed and measured the height of participating children at their respective preschool with staff present. An electronic digital scale with step-on technology was used to collect the weight to the nearest 0.1 kilograms, and a stable stadiometer was used to measure height. A 2-pound weight was weighed at each site for scale calibration before children were weighed. Per conventional guidelines, children were asked to remove jackets, shoes, and anything in their pockets.

### Measuring weight perception

Misclassification has consistently been defined as a discrepancy between how a parent describes their child’s weight versus what the child weighs [17–21]. Despite a commonality among what misclassification is classified as there have been varying measurements used to determine misclassification. The research has largely utilized three methods to assess the accuracy of parental classification: 1. Parental classification of weight on a Likert scale using categories such as underweight, normal, overweight, or obese [8, 22]. 2. Parental classification by selecting a picture that most accurately aligned with their child’s weight [8, 22] 3. Parental classification by asking to write how much they believed their child weighed [19].

As no one method has been identified as the most accurate, this study employed all three methods of assessment for misclassification. Parental assessments were then compared to the actual child weight and height using the BMI-for-age percentiles [23].

### Measuring HBM components

The three measures of perceived severity, perceived susceptibility, and perceived barriers were used as components of the HBM. Perceived severity was measured using the Obesity risk scale-10 (ORK-10), which is a 10-question scale that has true-false questions [24]. For perceived barriers, the 16-item Parental Self-Efficacy for Promoting Healthy Physical Activity and Dietary Behaviors in Children Scale (PSEPAD) [25] was used. Perceived susceptibility was measured using two separate questions regarding parental concern as well as obesity exposure (measured by one question). The questions were used in past research [26].

### Research questions and statistical analysis for each

#### General data analysis

Parents were asked to self-report their height and weight. The investigator-assessed all child heights and weights as previously described; the average parental weight was taken as a general health measure by self-report. The average parental weight was 164.50 (SD = 39.89) pounds. The average parental weight was self-reported in pounds, and their height was self-reported in inches. The investigator used the self-reported weight and height to assign each parent a BMI {weight (kg) / [height (m)]2} and a categorical label (underweight, healthy, overweight, and obese) based on CDC recommendations [27]. Standard CDC weight categories rank adult weight as follows: 18.5 or less is underweight, between 18.5 and 24.9 is a healthy weight, 25 to 29.9 is overweight, and 30 or above is obese.

Unlike adult BMI, child BMI is sex and age-specific, so it is not possible to categorize the average BMI as normal, overweight, or obese just by the numbers alone [28]. However, child BMI is reported with a corresponding percentile, which is used to classify weight status [28]. A normal weight percentile is between 5 and 85, while less than five is underweight, overweight is between 85 and 95, and obese is greater than or equal to 95. For analysis of the visual perception of child size, an artist rendering depicting weight as used [29].

The questionnaire also included various questions to help describe the study population and parental views. Previously explored factors were included as part of the questionnaire including child age [2, 30–33] and child gender [32–36]. Parental factors including exposure to health-related conditions [37], concern [30], and obesity-risk knowledge [37, 38]. These questions were reported in percentages to give valuable information about the study population.

#### Question one: Do the continuous ORK-10 scores predict parental child weight classification for the three methods of weight classification?

Logistic regression was used for this analysis. Variables include the use of the continuous ORK-10 scores and the categorical label representing child weight. This approach was planned as underestimation has also been a significant finding in the research. Hence, weight classification for the three methods of parental child weight assessment was also be broken into 1 = correct, 2 = underestimation, and 3 = overestimation. Correct classification was used as the referent category for the multinomial logistic regression.

#### Question two: What are the associations of perceived susceptibility (as measured by two separate parental concern questions) and obesity exposure (as measured by one exposure question) to the parental classification of preschool child weight as measured by the accuracy of parental child weight assessment for all three assessment methods?

Measures had not been developed to examine parental concern or obesity exposure, but questions related to these factors have been used in previous studies [30, 34]. As there was not a cohesive scale, each question was asked and answered by parents selecting a number from a 5-item Likert scale. The numbers corresponded to the level of concern about child weight. Each value was compared to the dichotomous measure of correct and incorrect to assess for potential associations. The two concern questions were used to provide descriptive data showing the spread of how parents rated their concern on the Likert scale. Again, association tests were used to determine if increased parental concern leads to more or less accurate classification of their child’s weight. The association of responses to the two Likert-type scales with the dichotomy of correct or incorrect for parental child weight classification was assessed for all three methods. A chi-square test and Cramer’s V were conducted.

Exposure was measured by categorizing depression, diabetes, heart disease, hypertension (high blood pressure), and hyperlipidemia (high cholesterol) as yes/no for the presence of family history (exposure). A chi-square test was run to compare the presence of any obesity-related disease (of the five asked about) categorized as yes/no to classification status (yes/no) to assess the association. A phi coefficient was reported to indicate the degree of association. The main 76 analysis for this variable was conducting a single test for obesity exposure base on a dichotomy of 1 = family history exposure and 0 = no exposure across the five family history questions.

#### Question three: Do perceived barriers as measured by Parental Self-Efficacy for Promoting Healthy Physical Activity and Dietary Behaviors in Children Scale (PSEPAD) correlate with and predict the parental classification of preschool child weight status as measured by the accuracy of parental assessment for all three assessment methods?

The 16-item PSEPAD scale assessed three factors: No. 1 is PSE for promoting healthy dietary behaviors, No. 2 is PSE for the limit setting of unhealthy dietary or physical activity behaviors in children, and No. 3 PSE for promoting healthy physical activity behaviors in children [25]. Only the total score was used for analysis in this study. The total score possible is 160 as parents are asked to select a rating from 1 to 10 scoring their confidence in performing the various measures. A higher score indicates increased self-efficacy for controlling child behaviors.

The PSEPAD was measured as a total score. A higher score was indicative of increased self-efficacy for control over child behaviors. This numeric score was compared with the parental classification of weight status, which was measured as a dichotomous variable of correct and incorrect. Logistic regression was used for these analyses and the point-biserial correlation was reported.

#### Question four: Does the weighted combination of parental self-efficacy gauged by the PSEPAD score and knowledge of obesity health risks (based on the ORK-10) predict the accuracy of parental classification (correct/incorrect) for all three assessment methods (Likert, visual, and reported weight)?

Parental assessment of weight was coded into three categories (1 = *correct*, 2 = *underestimation*, 3 = *overestimation*). The correct classification was listed as the referent category. These predictors of the outcome were performed to determine if PSEPAD and ORK-10 total scores could predict parental classification (0 = incorrect, 1 = correct). As previous literature showed that child sex, obesity exposure, and parent age [8] have been shown to potentially influence perception these variables were included in the regression analysis.

## Results

Stata 14.1 (Stata Corporation, College Station, TX, U.S.) and IBM SPSS Statistics 25 were used to analyze research data from the written surveys and investigator assessments. Between either the daycare sending surveys home with parents and the investigator handing out surveys, 415 surveys were distributed. A total of 198 of 415 surveys were returned, making the response rate 47.7%. Response rates where the investigator spoke with individuals and requested their participation had higher response rates ranging from 71.4% to 77%. One child had missing weight data as they were not available for height/weight measurement; thus, the parent and child were excluded from data analysis.

### Participant characteristics

Participant data and information related to each of three classification methods were reported in a previous article [39]. Most parents were female (n = 176, 11.1%), age 30–39 (n = 104, 52.8%), had an income of between $75,000 and $99,999 (n = 52, 26.9%), and had some college or an associate’s degree (n = 82, 41.4%). The data did include two grandparents who were the child’s primary caregiver and 22 fathers. The average age of participating children was 45.5 months (SD = 11.11) with a minimum age of 24 months and a maximum of 71 months. Table 1 breaks down characteristics of the children participating.

**Table 1:** Characteristics of participating children.

Child demographics	No. (%)
*S*e*x*	
Male	100 (50.5)
Female	98 (49.5)
Age	
2	31 (15.7)
3	58 (29.4)
4	87 (44.2)
5	22 (11.2)

**Fig 1:**
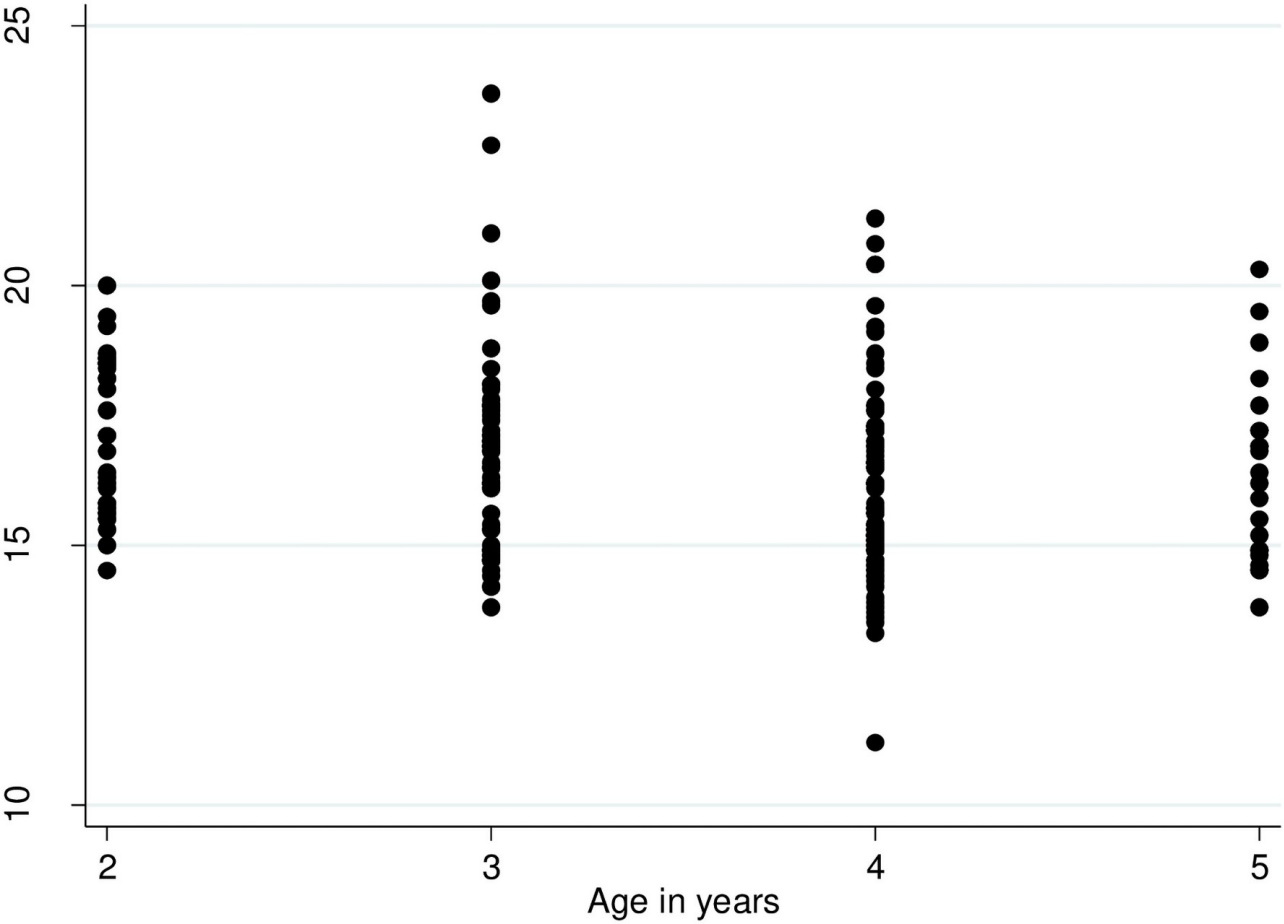
Changes in child BMI increase in age in years for the 197 child participants. This shows child BMI by age to visually describe how the BMI varied among participants.

Fig 1 also shows the change in child BMI with age change.

The average adult BMI was 27.20 (SD = 6.03), which is considered overweight by CDC guidelines. Other parent weight categories included underweight (n = 5, .03%), healthy weight (n = 98, 49.5%), and obese (n = 15, 7.6%). Children had an average BMI percentile was 61.75 (SD = 30.77), which is indicative of a normal weight. The breakdown across classifications for children was: underweight (n = 6, 3%), healthy weight (n = 118, 59.9%), overweight (n = 47, 24%), and obese (n = 26, 13%). The second-highest 181 number of children were classified as overweight (n = 47, 23.9%). Table 2 shows how child and parent weight status varies among the participants.

**Table 2:** Actual parent and child weight frequencies and percentages by weight category and by sex.

Category	Female	Male	Overall
*Child*	*Female (n = 97)*	*Male (n = 100)*	*Overall (n = 197)*
Underweight	3 (3.0)	3 (3.0)	6 (3.0)
Healthy weight	64 (66.0)	54 (54.0)	118 (60.0)
Overweight	20 (21)	27 (27.0)	47 (24.0)
Obese	10 (10.0)	16 (16.0)	26 (13.0)
*Parent*	*Female (n = 176)*	*Male (n = 22)*	*Overall (n = 198)*
Underweight	4 (0.02)	1 (0.05)	5 (0.03)
Healthy weight	88 (50)	10 (45.5)	98 (49.5)
Overweight	70 (39.8)	10 (45.5)	80 (40.4)
Obese	14 (0.08)	1 (0.05)	15 (7.6)

*Note*. Information is reported as number (%).

One child had a missing weight value.

Most parents (97.5%, n = 192) reported taking their child to a well-child visit each year, and 65.8% (n = 131) felt their child was healthier than other children. The sample of 188 children also had few chronic illnesses with most (87.4%, n = 174) with none, 8.5% (n = 17) with one, and 3.5% (n = 7) with two. Outside of regular well-child exams, 20.1% (n = 40) had no MD visits, 38.2% (n = 76) saw a doctor once, 24.1% (n = 48) saw a doctor twice, and 17.1% (n = 34) saw a doctor three or more times in the last year. Parents were also asked if a health care provider had ever mentioned their child being overweight or obese. While 98.5% (n = 196) parents said no, 1% (n = 2) said yes and one parent did not answer (0.5%). The two parents, who said yes, had a child who was classified as obese based on CDC guidelines. According to the weight classifications of children, 37.2% (n = 73) should have been told their child was either 196 overweight or obese. Parents were also asked how likely they would be to do something about weight issues. Of the 198 total parents, 76.4% (n = 152) said they would be extremely likely, and 17.6% (n = 35) were moderately likely to do something about their child’s weight issues if a health care provider informed them there was a problem.

#### Misclassification of child weight

As a basis for the three questions, first the parental ability to classify child weight needed to be assessed. The three reporting methods had varying degree of accuracy with perception of child weight: Likert method 53.3%, weight-reporting method 50.3%, and pictorial method 35.9%. Kappa values showed that with both the pictorial method (κ = -.028, p = .42) and Likert scale method (κ = -.032, p = .37) parental agreement with actual child weight was not significantly better than chance alone. Meanwhile, there was a slight statistically significant agreement observed with the weight-reporting method and actual child weight (κ = .21). Parents were better able to determine their weight as demonstrated by the agreement between the Likert description selected and their BMI determined by self-reported height and weight (κ = .43, p = .05). A biserial point correlation between child weight and correct classification by Likert scale showed that as weight decreased, parents’ inaccuracy increased significantly, rpb = -.18, 95% CI [.04, -.31], p = .011.

Table 3 describes the agreement between parental classification and actual child weight.

**Table 3:** The agreement between parent’s descriptions of the child’s perceived weight status and the child’s actual weight status based on CDC guidelines at age 2 to 5.

Parental report of weight status	Child’s actual weight status, *n*			
	Underweight	Healthy	Overweight	Obese
*Likert scale*, (*n* = 198)				
Underweight	2	17	1	0
Healthy	4	98	45	21
Overweight	0	4	1	5
Obese	0	0	0	0
*Weight in pounds*, *(n = 183)*				
Underweight	2	24	4	1
Healthy	3	77	21	10
Overweight	0	4	9	6
Obese	1	2	10	9
*Pictorial*, (*n* = 198)				
Underweight	3	50	12	6
Healthy	3	64	31	19
Overweight	0	4	4	1
Obese	0	0	0	0

*Note*: CDC is Center for Disease Control.

#### Question one: Do the continuous ORK-10 scores predict parental child weight classification for the three methods of weight classification?

**Fig 2:**
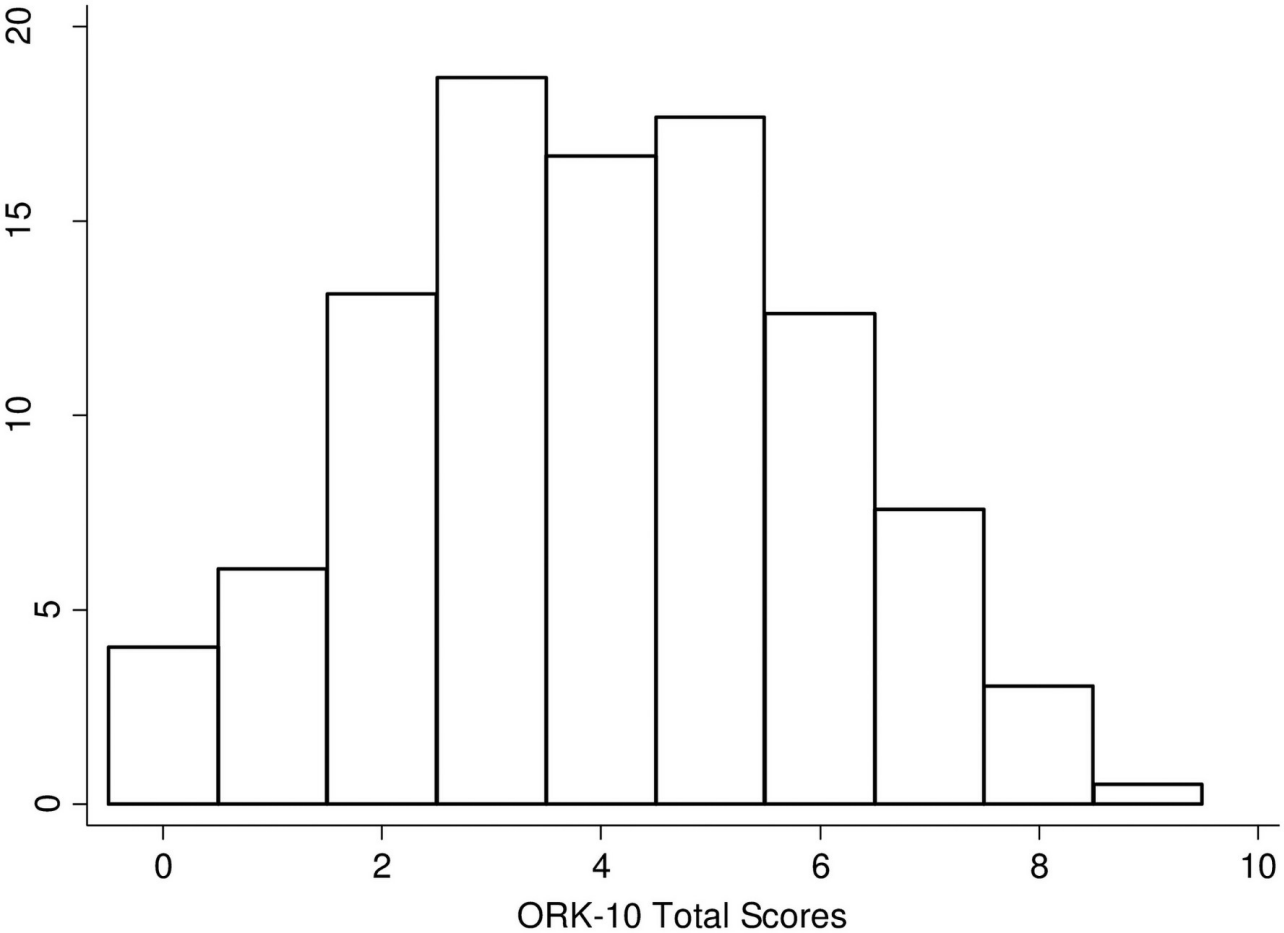
Display of 198 parent scores on the ORK-10 scale that examines knowledge of obesity knowledge related to health risks. The histogram displays the percent of parents meeting each total score.

The ORK-10 is a 10-item test that tests knowledge of obesity risks. Participating parents had scores ranging from 0 to 9 (M = 4.01; SD = 1.97). The histogram showing these results is displayed in Fig 2.

Initial correlation analyses showed that the ORK-10 score had a small, but significant relationship with parental ability to correctly classify child weight with the Likert scale, rpb = .17, p = .015. This relationship indicates a small tendency for higher ORK-10 scores to relate to accuracy in the classification of child weight. However, there was no statistical significance found with the total ORK-10 score and either pictorial classification, rpb = .10, p = .16, or parental classification by report of child weight, rpb = .07 p = .32.

Of the 183 parents 237 who provided a reported weight for their child, 91 were incorrect. This incorrect classification included 72 (39.3%) who underestimated child weight and 19 (10.4%) who overestimated their child’s weight. The binary logistic regression was statistically significant, -2 Log Likelihood = 72.77, χ2(2, n = 183) = .181, *p* = .018. The Nagelkerke pseudo R2 = .006 indicates the model accounted for 0.6% of the total variance in classification. Neither underestimation of child weight nor overestimation of child weight by parents proved to be statistically significantly predicted in the model. This model was able to predict underestimation correctly only 4.2% of the time, and no cases of overestimation were accurately predicted.

Neither the Likert method nor the pictorial method could be examined with multinomial regression as the overestimation of child weight had too few values. With the Likert method, only one parent overestimated child weight (n = 1, 0.5%). The rest of the parents were either correct (n = 103, 52.3%) or underestimated child weight (n = 93, 47.2%). Meanwhile, 126 people misclassified weight using the pictorial method. Of those who misclassified, 60.9% (n = 120) underestimated weight and 3.0% (n = 6) overestimated weight. Therefore, binary logistic regression was used to predict parental accuracy in classification as (0 = incorrect, 1 = correct) with weight classification and their total ORK-10 score.

The mean ORK-10 score for parents was M = 4.01 (SD = 1.97). Of the 197 parents who provided a Likert rating for their child, 92 (46.7%) were incorrect about their child’s weight. The binary logistic regression was statistically significant, -2 Log Likelihood = 266.28, χ2(5, n = 256) = .181, *p* = .017. The Nagelkerke pseudo R2 = .040 indicates the model accounted for 4% of the total variance in classification. The Wald test showed that ORK-10 scores were a statistically significant predictor (*p* = .017) of correct classification. Parents who were correct in classification scored 0.181 higher than those who were incorrect. For every one-unit score increase in the ORK-10 score, parents were 1.2 times more likely to have a correct classification of weight. This information is presented in Table 4.

**Table 4:** Predictors of correct classification using the parent report of child weight with the Likert scale.

Model	*B*	SE-*B*	Wald	*Df*	Exp(*B*)	95% CI Exp(*B*)
Intercept	-0.592	0.334	3.15	1		
Total ORK-10	0.181	0.076	5.74*	1	1.198	[1.03, 1.39]

*Note*. The correct group was the target outcome.

*p < .05.

ORK-10 = Obesity Risk Scale.

No significance was found with the ORK-10 scores and pictorial parental classifications of correct and incorrect. The binary logistic regression had -2 Log Likelihood = 255.42, χ 2 (5, n = 197) = .111, p = .148. The Nagelkerke pseudo R 2 = .015 indicates the model accounted for 1.5% of the total variance in classification.

#### Question two: What are the associations of perceived susceptibility (as measured by two separate parental concern questions) and obesity exposure (as measured by one exposure question) to the parental classification of preschool child weight as measured by the accuracy of parental child weight assessment for all three assessment methods?

**Fig 3:**
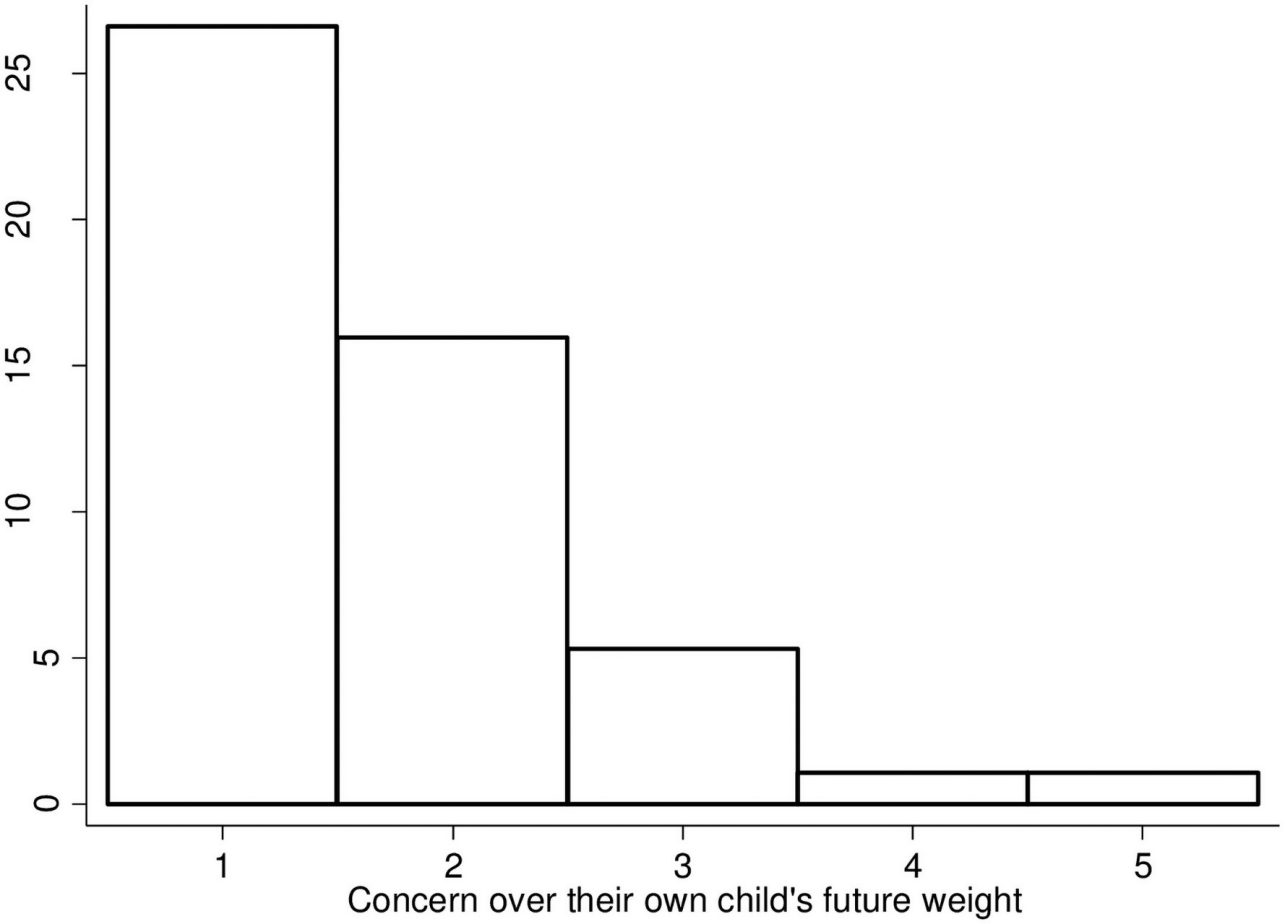
Visual depiction of parental concern in overweight children. This bar chart shows the level of concern the 47 parents of overweight children have regarding their child’s future weight status. The concern level ranges from unconcerned (1) to very concerned (5). Most parents were either unconcerned (1) or a little concerned (2) regarding the future weight status of their already overweight child.

Parental concern over the future weight status of their child had a positive skew as 59% of parents (n = 118) selected being “unconcerned.” This remained true even for parents with overweight children (n = 26, 22.0%) and obese children (n = 15, 12.7%). One parent of an overweight child and one parent of an obese child reported the highest rating of being “very concerned” about their child’s future weight. The second most selected category was being “a little concerned” about their child’s weight (n = 59), which was selected by 2 parents of underweight children, 37 parents of normal-weight children, 15 parents of overweight children, and 5 parents of obese children. These first two categories comprised 89.4% of responses (n = 177) regarding the concern of their own child’s future weight status. The trend of positively skewed data remained true even with overweight children, where 25 of 47 parents of overweight children reported being “unconcerned” about the future weight status of their children. Fig 3 visually depicts how parents of overweight children answered this question.

The answers for concern over children’s future weight status in the nation were more evenly spread with 28.8% (n = 57) parents selecting being “concerned,” which was option three of five on the Likert scale. The remaining choices comprised “unconcerned” at 10.1% (n = 20), “a little concerned” at 24.2% (n = 48), “fairly concerned” at 18.7% (n = 37) and being “very concerned” at 18.2% (n = 36). This pattern of equal spread remained true for parents of healthy weight children. Parents of overweight children had a negative skew to how they answered this question. Parents of 14 of the 47 overweight children (38.9%) reported being “very concerned” with the children in the nation’s future weight while only 6 (12.7%) reported being “unconcerned.”

How concerned parents were with the weight of their own child versus the weight of children in the nation as compared to parent ability to classify weight using the three methods. Cramer’s ϕ was used to determine the level of association between the reported levels of concern expressed with each classification method. No association was found between either the level of concern of their own child’s future or concern over the future weight of children in the nation by any classification method. Using parent report of pounds, the result was Cramer’s ϕ = .12, p = .582, for their own child and Cramer’s ϕ = .15, p = .416 for children in the nation. classification method had a Cramer’s ϕ = .14, p = .390, for their own child and Cramer’s ϕ = .08, p = .885, for children in the nation. Lastly, the pictorial classification method had a Cramer’s ϕ = .10, p = .724, for their own child and Cramer’s ϕ = .17, p = .208, for children in the nation. Table 5 shows the breakdown of both concern for one’s own child and concern regarding the weight of children in the nation is broken down by correct and incorrect classification status for all three methods.

**Table 5:** Comparison of concern of weight of own child versus national weight of children by classification status.

Level of Concern	Method and Classification Status			
	*Pictorial Method*			
	*Correct (n = 105)*		Incorrect (*n* = 92)	
	*Own Child*	*Nation*	*Own child*	*Nation*
Unconcerned	40	7	77	13
A little concerned	22	15	38	33
Concerned	5	21	6	36
Fairly concerned	1	19	3	18
Very concerned	3	9	2	26
	Likert Method			
	*Correct (n = 71)*		Incorrect (*n* = 126)	
	*Own child*	*Nation*	*Own Child*	*Nation*
Unconcerned	62	7	55	11
A little concerned	34	15	26	22
Concerned	3	21	8	24
Fairly concerned	3	19	1	18
Very concerned	3	9	2	17
	Weight Method			
	*Correct (n = 92)*		*Incorrect (n = 92)*	
	*Own child*	*Nation*	*Own child*	*Nation*
Unconcerned	57	12	54	8
A little concerned	28	26	24	20
Concerned	3	27	8	25
Fairly concerned	2	12	2	20
Very Concerned	2	15	3	18

Parents were also asked about whether they had a family history of overweight as well as obesity. If parents reported yes, then they were determined to have exposure to either overweight or obesity. Most participants (74.2%, n = 147) had exposure to a family history overweight status while a smaller number (40.4%, n = 80) had exposure to a family history obesity status. Of the 147 who had had a family history of overweight status, 54.4% also had a family history of obesity exposure, which was a statistically significant association shown through a chi-square test. Additional chi-square examinations were conducted to determine if a family history of overweight status was associated with classification ability using the three methods. There were no statistically significant associations found. Three additional chi-square tests of association were done to see if a family history of obesity affects classification ability by the three methods. The only method that had significance in the association was with the pictorial classification method and obesity exposure. This showed that 34.9% (n = 44) of those who misclassified their child’s weight had a family history of obesity exposure versus 49.3% (n = 35), who correctly classified weight having a family history of obesity exposure.

The only method that had significance in the association was with the pictorial classification method and obesity exposure. This showed that 34.9% (n = 44) of those who misclassified their child’s weight had a family history of obesity exposure versus 49.3% (n = 35) who correctly classified weight having a family history of obesity exposure. The chi-square was statistically significant, χ 2 (1, N = 197) = 3.91, p = .048. With the weight-reporting method, 67% (n = 61) of those who misclassified child weight had exposure to obesity while 44.6% (n = 41) who correctly classified weight had exposure to obesity. The chi-square test was not statistically significant, χ 2 (1, N = 183) = 2.394, p = .122. Using the Likert reporting method, 63% (n = 58) of those who misclassified child weight had exposure to obesity while 42.8% (n = 45) who correctly classified weight had exposure to obesity. The chi-square was not statistically significant χ 2 (1, N = 197) = .711, p = .399.

#### Question three: Do perceived barriers as measured by Parental Self-Efficacy for Promoting Healthy Physical Activity and Dietary Behaviors in Children Scale (PSEPAD) correlate with and predict the parental classification of preschool child weight status as measured by the accuracy of parental assessment for all three assessment methods?

The mean of PSEPAD scores was 125 (SD = 20.71), with a total possible of 160. The scores varied from a low of 63 to a high of 160 (25th percentile = 113; 75th percentile = 140). The skew of the scale was -0.59, and the kurtosis was 2.92. A point-biserial correlation was also used to examine the relationships. No significant correlations were found between the total PSEPAD score and correct or incorrect parental child weight classification by any method. Correlation of parent weight report as correct and incorrect compared to total PSE was not statistically significant, rpb = -.00, *p* = .98, as was classification by pictorial method, rpb = .06, *p* = .43. This shows there were no significant correlations between parental self-efficacy scores and the correct classification of their child’s weight. Of the three methods, the highest correlation was identified between the correct classification by Likert description and total PSE score, rpb = .11, *p* = .12, but the relationship was not statistically significant. There was also no statistical significance found for the ability of PSEPAD scores to predict pictorial classification as (0 = incorrect, 1 = correct).

Of the 183 parents who provided a reported weight for their child, 92 (50.5%) were correct and 90 were incorrect. This incorrect classification included 71 (39%) who underestimated child weight and 21 (10.4%) who overestimated their child’s weight. The multinomial logistic regression was not statistically significant, -2 Log Likelihood = 213.82, χ2(2, n = 182) = 1.02, *p* = .60. The Nagelkerke pseudo R2 = .007, indicating the model accounted for less than 1% of the variance in classification.

#### Question four: Does the weighted combination of parental self-efficacy gauged by the PSEPAD score and knowledge of obesity health risks (based on the ORK-10) predict the accuracy of parental classification (correct/incorrect) for all three assessment methods (Likert, visual, and reported weight)?

First, a multiple binomial logistic regression with two predictors of the binomial outcome of correct or incorrect was performed to determine if PSEPAD and ORK-10 total scores could predict parental classification (0 = incorrect, 1 = correct). Table 6 shows regression coefficients, Wald tests, the odds ratio, and the 95% confidence interval for regression coefficients for each predictor.

**Table 6:** Binary logistic regression results of multiple predictors of correct classification of child weight with Likert classification by parents.

Model	*B*	SE-*B*	Wald	*Df*	Exp(*B*)	95% CI Exp(*B*)
Intercept	2.26	1.001	5.09*	1		
PSEAD	-0.013	0.007	3.215	1	0.987	[0.97, 1.00]
ORK-10	-0.195	0.077	6.444*	1	0.822	[0.71, 0.96]

*Note*. Correct classification was the target outcome group.

*p < .05.

ORK-10 = Obesity Risk Scale.

PSEAD = Parental Self-Efficacy for Promoting Healthy Physical Activity.

The logistic regression was statistically significant, -2 Log Likelihood = 239.86, χ 2 (1, n = 197) = 5.30, *p* = .021. The Nagelkerke pseudo R 2 = .034, indicating the model accounted for 3.4% of the variance in classification. The Wald test showed that the ORK-10 score was a statistically significant (*p* = .011) predictor for correct classification of child weight by Likert classification. For every one-unit increase in the ORK-10 score, parents were 0.82 times as likely to correctly predict their child’s weight when controlling for the PSE score. Meanwhile, the Wald test approached significance for PSEPAD to be a predictor of correct classification (*p* = .07). This showed that for every unit increase in the ORK-10 score, parents were 0.987 times = more likely to correctly predict their child’s weight when controlling for the PSEPAD score.

Correct classification was not able to be predicted by either of the other two methods of child weight classification. A multiple binary logistic regression with five predictors of the binomial outcome of correct or incorrect was performed to determine if these combined factors could predict parental classification. The logistic regression was statistically significant, -2 Log Likelihood = 251.83, χ 2 (5, n = 197) = 17.625, *p* = .003. The Nagelkerke pseudo R 2 = .115, indicating the model accounted for 11.5% of the variance in classification. The Wald test showed that the ORK-10 score, the parental self-efficacy (PSEPAD) score, and child sex were also significant predictors for correct classification of child weight by Likert classification.

For every one-unit increase in the ORK-10 score, parents were 1.26 times as likely to correctly predict their child’s weight when controlling for the PSEPAD score, obesity exposure, child sex, and parental age. Meanwhile, the Wald test also confirmed the PSEPAD score to be a predictor of correct classification. This showed that for every unit increase in the PSEPAD score, parents were 1.016 more likely to correctly predict their child’s weight when controlling for the other factors. Table 7 shows regression coefficients, Wald tests, the odds ratio, and the 95% confidence interval for regression coefficients for each predictor.

**Table 7:** Binary logistic regression results of multiple predictors of correct classification of child weight with Likert classification by parents.

Model	*B*	SE-*B*	Wald	*Df*	Exp(*B*)	95% CI Exp(*B*)
Intercept	-3.917	1.342	8.512**	1		
Child Sex	0.723	0.303	5.676*	1	2.061	[1.14, 3.74]
PSEPAD	0.016	0.008	4.566*	1	1.016	[1.001, 1.032]
ORK-10	0.229	0.083	7.625**	1	0.822	[0.71, 0.96]
OE	0.217	0.313	0.480	1	1.243	[0.672. 2.296]
Age	-0.028	0.234	0.015	1	0.972	[0.614, 1.538]

*Note*. Correct classification was the target outcome group.

OE equals obesity exposure.

ORK-10 = Obesity Risk Scale.

PSEAD = Parental Self-Efficacy for Promoting Healthy Physical Activity.

Age is parental age.

*p < .05,

**p < .01.

Correct classification was not able to be predicted by either of the other two methods of child weight classification.

## Discussion

To the best of our knowledge, this is the first study that compares the three commonly used measures for parental to components of the HBM model (perceived severity, perceived susceptibility, and knowledge). Results showed that each method of judging parental perception is associated with inaccurate classification: 46.7% for Likert, 63.6% for pictorial, and 49.7% for the weight-reporting method. The main goal of this study was to identify if the HBM components could predict weight misclassification.

The general results of the ORK-10 (*M* = 4.01; *SD =* 1.97) are similar to others who looked non-experts (*M =* 4.81; *SD =* 1.97), which was part of initial testing for the scale [6]. Our results show the score on the ORK-10 scale did have a small, positive relationship with the parental ability to classify weight using the Likert scale. No research was identified showing usage of this score as a predictive element for classification, though the results do appear congruent with other research that shows obesity risk knowledge is linked to weight misclassification in pregnant mothers [40].

Regarding the second question of concern, our results showed that regardless of the child’s current weight status, parents were largely unconcerned about the child’s future weight. The results of 89.4% of parents either reported being “unconcerned” or “a little concerned” about their child’s future weight contrasts with other study findings. A previous study found that parents who did not correctly classify their child’s weight status still had a higher chance of being concerned about their child’s future weight [30]. Meanwhile, a second study identified that 46% of mothers were concerned about their child becoming overweight in the future and this concern was correlated to measures of BMI, skinfold score, and waist circumference [36]. Both identified studies that looked at the relationship of concern and parental classification of child weight were conducted in the United Kingdom with larger sample sizes than our study. It could be that a difference in concern exists solely because of population differences. Obesity prevalence was 39.8% and affected 93.3 million US adults from 2015 to 2016 [1]. The United Kingdom of Great Britain and Northern Ireland, where the two comparison studies were conducted, had obesity rates of 26.9% in 2008 [41] versus 33.7% in the United States in 2008 [1]. These weight differences could also change views on current and future weight status and its importance.

Another component of our second research aim was that of overweight or obesity exposure. Most of our sample reported having family members who would be considered overweight at 74.2%, while fewer reported obesity in the family (41.9%). Having a family history of overweight status did not prove significant to the ability to correctly classify child weight. In fact, no significant relationship was found between an overweight history and any of the three classification methods. Likewise, having a family history of obesity exposure had no significant relationship to correct weight classification using the Likert or weight-reporting method. However, with the pictorial method, parents who had obesity exposure had a significantly higher chance of correctly classifying weight.

This study’s findings mirrored others where parents had little to no concern over their child being overweight or obese [42–44]. A previous review posited that a large amount of misclassification may be linked to a distorted parental understanding of what an overweight child looks like due to popular media only using severely obese children as examples [8]. This same concept could explain why parents are not concerned with their child’s weight status. If they only view higher weights as a problem, then their view of when to be concerned as well as what is an abnormal weight could be distorted.

Meanwhile, our results showed that parents who identified overweight family members in their family history had no better perception of weight than others. It was only when people identified obesity in their family history that perception improved. Parents who had a family history of obesity were found to classify weight more accurately via the pictorial method in this dissertation study. This finding is important because it showed that only large variations from normal weight are linked to changes in perception, and this was only seen when parents selected weight representations on a visual scale. It could mean that parents with that exposure to obesity have a more accurate visual template than the generalized misrepresentations seen in mainstream media and society. Past research provides evidence that multi-level modeling can affect how children even see themselves when exposed to overweight and obesity in both their home and their school environment [45]. Children who see overweight and obese people in their environment regularly thus can develop inaccurate perceptions of what constitutes appropriate weight [45]. While our study focused on parental views only, further exploration into this idea with children as well is warranted.

Our third question dealt with parental self-efficacy and utilized the PSEAD scale [25]. Parental results were negatively skewed with at the 25th percentile equaling 113 and at the 75th percentile equaling 140 (*M* = 125, SD = 20.71). The results showed that PSEAD scores could not predict parental classification ability, which was previously seen in past research [31]. Both these results contradict other findings where lower parental self-efficacy scores were related to higher body mass index in children [46, 47]. The results are thus divided on whether parental efficacy is a factor, and this indicates further exploration is needed to clarify this issue. It could be that self-efficacy is best used when tailoring appropriate interventions for weight maintenance and loss. After all, the connection of parental self-efficacy and healthy behaviors like eating fruits and vegetables and increased physical activity in children has been well-established [46, 48–53].

The split results could also be related to how self-efficacy is being measured and the size/variability of the population included.

The last questions dealt with the examination of how the combined effects of the proposed HBM items might affect the prediction of parental perception. Results in this study regarding perceived barriers (examined through the PSEPAD) and perceived severity (examined by the ORK-10) show a significant relationship to parental classification with the Likert method. Further, the results of low ORK-10 scores and moderate PSEPAD scores are below optimal thresholds and thus have the potential for improvement.

This study offers new information that shows that parents have the most accuracy with classifying weight using the Likert method. The ORK-10 scale and PSEPAD showed the predictive ability of the Likert classification method. As these were measures for perceived barriers and perceived severity from the HBM, this identifies a potential link to understand parental perceptions of child weight and a framework for future interventions. Each of these items shows a modifiable component in parents that can be linked to promoting healthy behaviors in children. It is plausible to conclude that increased knowledge and parental self-efficacy toward healthy behaviors may improve the accuracy of how parents perceive child weight and health.

This study also showed that parent concern about weight (perceived susceptibility) was not linked to classification ability. Results showed parents were unconcerned about their child’s future weight, even though 36.9% of children were already overweight or obese and despite that weight issues were common in the reported family histories. So, while this component of the HBM was not connected to classification, it is still of interest. At the very least, these findings should be concerning for health care providers because many parents showed a general lack of awareness about their child’s accurate weight. Further, if parents do not recognize weight is a problem, the likelihood of them acting on the child’s behalf is not good.

While not part of the three examined components of the HBM, there was also an important finding regarding parental health behaviors. Parents reported attending yearly well-child checks and noted they would be willing to intervene if a health provider told them that their child had a weight problem. These findings, coupled with the lack of concern, suggest child weight is an important concept that should be discussed with parents. Considering the reported willingness to intervene and the regular visits for check-ups, it is also very concerning that only two parents reported being notified by a healthcare provider that their child had an issue with weight. This is not in line with the number of overweight and obese children in the sample. Given that most parents reported taking their child to the yearly appointment, more parents should have been told of abnormal weights.

If parents are supportive of being told their child has weight issues and would be inclined to intervene as this study’s findings suggest, then communication needs to be improved between health care providers and parents. This finding points to the need for nurses or other healthcare providers to talk to parents about their child’s weight and tell them when a weight problem may be developing. Given varied efficacy levels toward promoting healthy behaviors and limited knowledge of weight-related health risks, it is important to gauge how parents fare in these categories. It may be important to intervene relating to these factors for parents to understand why child weight is important and how they can affect it. Addressing weight issues requires that providers can discuss issues openly and honestly with parents and that there be a collaborative approach to take action on behalf of children. Parents must perceive that a child has a problem and identify the risks associated with weight.

Considering the known, significant consequences for childhood obesity, educational interventions teaching children and parents’ habits for healthy lifestyles is imperative and can help minimize the risks associated with increased weight [54]. Health teaching is recognized to help people understand influential behaviors and replace them, when necessary, with new, more appropriate behaviors [55]. In the preschool population, parental recognition of child body weight is imperative to inspire behavior modifications in the home [43]. This study corroborated previous findings showing that parents have a difficult time identifying appropriate body weight in their children, and that this problem is significantly exacerbated when the child is overweight or obese [33, 39, 56, 57] Research has shown that a parent’s perception of weight status can influence both parents and their child’s healthy behaviors [5].

### Limitations and generalizability

This study had multiple limitations affecting generalizability. First, the use of a convenience sample limits the generalizability as parents who agreed to participate may have different sentiments about weight in preschool children than those who did participate. Reasons for not participating were not tracked for the 217 people who did not return surveys. Most of these non-returned surveys resulted when surveys were sent home with children and parents were asked to return if they were willing to participate. Therefore, why they chose not to participate could not be identified. Second, the participants in this sample also were mostly white females with at least some college. While the racial and ethnic composition was similar to that of Utah and the areas where sampling occurred, it is not comparable to other areas of the state and country. This study also focused on parents of preschool children only so the results may not represent parents of infants or adolescents. These findings affect the ability to generalize results, and, therefore, they cannot be said to apply to all or even most parents of preschool children.

The study utilized a descriptive, cross-sectional approach that can only capture weight and opinions at only one point in time. Looking at misclassification at just one point may not be representative of the fluid nature of thinking and perception. The impact of life events and experiences, as well as changes in knowledge and health, could vary over time and thus affect perception. However, given the limited findings regarding how the HBM model relates to the perception of child weight, the cross-sectional approach was appropriate. There is also a concern regarding a parental report of weight. The study questionnaire asked parents to report the weight of their preschool child in pounds. There was not a statement that asked parents to report this without weighing their child. Also, there was not a question that asked if the parents did weigh their child prior to answering this item. This means that accuracy or inaccuracy in the parent report of weight cannot be separated from whether the parent used a scale or not. It could be that the large percentage of parents who were accurate in reporting weight, were accurate only because they weighed their child.

## Conclusions

The combined model used in this study provided new information about the connections between variables that warrants further exploration. The original model indicated there were only direct effects of the HBM principles. However, this is not accurate. Results identified an independent effect of perceived barriers (self-efficacy) and perceived severity (knowledge) on the parent’s ability to assess child weight accurately. Further, child sex was also significantly associated with the parent’s ability to assess their child weight accurately, even after controlling for other covariates in the model. Further, child sex acts as a mediator that suppresses part of the ORK-10 effect and is unrelated to its acting as a suppressor of the variance of the PSEPAD’s relationship to Likert accuracy. Both barriers and severity were found to be significantly related to parental classification ability by the Likert method. It is therefore important to further explore these connections with additional research. As these components are integral in the HBM model, finding a way to incorporate these items into intervention and prevention strategies and gauge their impact on child weight/parental awareness is something that should be explored.

Further, given the Likert method having the most accuracy by percentages obtained, the results of the study and the connections among classification ability, PSEPAD score, and ORK-10 scores are important. Both knowledge and PSEPAD scores are easy, quickly testable measures. These items are also grounded in HBM principles that suggest perceived severity, a component linked to knowledge in this study, and perceived barriers, which were linked to efficacy, are important components needed for health behavior change. The link established through this study with perceived severity and perceived barriers to appropriate recognition suggests that these items are also related to the ability to recognize a problem as demonstrated by the correct classification of weight. Health care providers must be aware of not only the child’s weight but also factors that affect recognition and understanding of what weight means.

Both the PSEPAD and the ORK-10 may provide a useful tool when crafting interventions or prevention strategies as the PSEPAD shows how confident parents are with specific behaviors that support healthy weight and the ORK-10 shows how much knowledge parents have of how weight relates to health risks. This is something that could be looked at in future research to see if the scale can help provide useful information to frame intervention or prevention efforts.
